# Single-Cell Proteins Obtained by Circular Economy Intended as a Feed Ingredient in Aquaculture

**DOI:** 10.3390/foods11182831

**Published:** 2022-09-13

**Authors:** Antia G. Pereira, Maria Fraga-Corral, Paula Garcia-Oliveira, Paz Otero, Anton Soria-Lopez, Lucia Cassani, Hui Cao, Jianbo Xiao, Miguel A. Prieto, Jesus Simal-Gandara

**Affiliations:** 1 Nutrition and Bromatology Group, Department of Analytical Chemistry and Food Science, Faculty of Science, Universidade de Vigo, E32004 Ourense, Spain; 2 Centro de Investigação de Montanha (CIMO), Instituto Politécnico de Bragança, Campus de Santa Apolonia, 5300-253 Bragança, Portugal; 3 Instituto de Investigaciones en Ciencia y Tecnología de Materiales (INTEMA, CONICET), Colón 10850, Mar del Plata 7600, Argentina

**Keywords:** single-cell-protein, aquaculture, circular economy, sustainable, residues

## Abstract

The constant increment in the world’s population leads to a parallel increase in the demand for food. This situation gives place the need for urgent development of alternative and sustainable resources to satisfy this nutritional requirement. Human nutrition is currently based on fisheries, which accounts for 50% of the fish production for human consumption, but also on agriculture, livestock, and aquaculture. Among them, aquaculture has been pointed out as a promising source of animal protein that can provide the population with high-quality protein food. This productive model has also gained attention due to its fast development. However, several aquaculture species require considerable amounts of fish protein to reach optimal growth rates, which represents its main drawback. Aquaculture needs to become sustainable using renewable source of nutrients with high contents of proteins to ensure properly fed animals. To achieve this goal, different approaches have been considered. In this sense, single-cell protein (SCP) products are a promising solution to replace fish protein from fishmeal. SCP flours based on microbes or algae biomass can be sustainably obtained. These microorganisms can be cultured by using residues supplied by other industries such as agriculture, food, or urban areas. Hence, the application of SCP for developing innovative fish meal offers a double solution by reducing the management of residues and by providing a sustainable source of proteins to aquaculture. However, the use of SCP as aquaculture feed also has some limitations, such as problems of digestibility, presence of toxins, or difficulty to scale-up the production process. In this work, we review the potential sources of SCP, their respective production processes, and their implementation in circular economy strategies, through the revalorization and exploitation of different residues for aquaculture feeding purposes. The data analyzed show the positive effects of SCP inclusion in diets and point to SCP meals as a sustainable feed system. However, new processes need to be exploited to improve yield. In that direction, the circular economy is a potential alternative to produce SCP at any time of the year and from various cost-free substrates, almost without a negative impact.

## 1. Introduction

The world population continues to increase, and according to mathematical models, it will reach 10 billion people by 2050 [1]. This increment entails a series of associated problems, being one of them the ability to produce food with an acceptance level of safety for the entire population. The current models foresee that this demand can be satisfied but lead to great environmental impact. To avoid this negative side effect, developing new sustainable food production systems that allow healthy diets from a nutritional composition point of view are vital in our society [2]. For achieving these goals, current food systems require important transformations; among them, it is needed to provide alternative sources of protein. In the past few years, there has been an increase in fish and seafood production. Within this sector, aquaculture has grown faster than any other animal protein sector (7% annual growth rate over the past two decades versus 4% for poultry and 3% for fisheries), probably due to its lower feed conversion ratio (1.1–1.6 kg of feed per kg of edible fish) when compared to terrestrial species (1.4–1.8 for poultry, 2.6–4.4 for pork, and 3.5–9 for beef) [3,4,5,6,7]. In this sense, animal protein consumption from fisheries, including seafood, wild-catch, and aquaculture, reached 179 million tons in 2018 and it is expected to continue rising. If this tendency is maintained, the aquaculture production rate is expected to represent a higher percentage than captures [5,6]. One of the benefits of this increase in aquaculture production is countering the slow supply of caught fish in a sustainable way, as it may help to stop the depletion and promote the recovery of natural populations by reducing the catch of wild fish. In 2012, a study estimated that the global recovery of fisheries could generate increases in abundance (56%) and fish yields (up to 40%) [8]. Nevertheless, these optimistic expectations have not been achieved by 2022, with the current scenario showing up to 48% of individual stocks remaining below biomass targets and 40% exploited above sustainable rates [9]. Therefore, longer temporal windows are still needed to increase and improve the productivity of aquaculture and reduce the impact of the wild catches. For this, it is crucial to solve other drawbacks of aquaculture, such as the improvement of the management of land, water, feed, energy, and disease control, as well as minimizing water pollution [10].

Regarding feed, it is essential to understand aquaculture species’ feed requirements to develop enhanced and reformulated diets [11]. Indeed, to have optimal fish production, it is necessary to design specific diets that meet the demands of each target species, thus avoiding impaired growth and diseases [12,13]. Among the macronutrients considered for developing successful diets, proteins are key organic molecules. Nitrogen is a relevant element of proteins and essential for all living beings, since it is the main constituent of vital compounds such as amino acids, enzymes, nucleoproteins, nucleic acids, chlorophylls, etc. Therefore, nitrogen and its derivates are present in most of the metabolites excreted by living beings, especially some animal species such as birds or fishes. It is estimated that fish from aquaculture converts 20–40% of feed nitrogen into biomass and the rest is excreted as ammonia or ammonium, depending on the pH [14,15].

The FAO estimated that carnivorous species has requirements of 40–55% dietary protein while freshwater omnivorous and herbivorous species require levels of 30–40% [5]. Fish diets typically contain between 20 and 55% crude protein, with carnivorous species showing the highest requirements, mainly between 40 and 55%, although some authors point towards 35–60% [16,17]. This high amount of animal protein means that diets represent 40–70% of the aquaculture production costs [17]. This is one of the main controversies of this productive system and even becomes a larger drawback with the expected increment in the feed requirements up to 37.4 million tons by 2025 [18]. Therefore, it is urgent to show alternative protein sources to reduce the high animal protein dependence of this industrial sector. In this sense, the most relevant choices are plant-based ingredients, insect meals, food waste, and microbial and macroalgae biomass [17]. In this respect, in 2017, the European Commission drew up a law on the environmental and economic sustainability issues of aquaculture (Regulation 893/2017). This regulation allows the use of seven insect species in fish feed, being the most promising yellow mealworm (*Tenebrio molitor*), black soldier fly (*Hermetia illucens*), and common house fly (*Musca domestica*). All these species are a potential alternative as feed thanks to their mass rearing, promoting the concept of a circular economy and zero waste [19]. However, even though the use of some insect meals is expected to promote environmental benefits, it would increase the feeding costs (high market prices of insect flour and less convenient feed conversion ratio than that of fish meal), and so it would not fit with the current economic interests of the aquaculture industry. Hence, additional efforts are still required to find cost-effective ways to introduce alternative diets, ensuring both economic and environmental sustainability [20].

**Figure 1:**
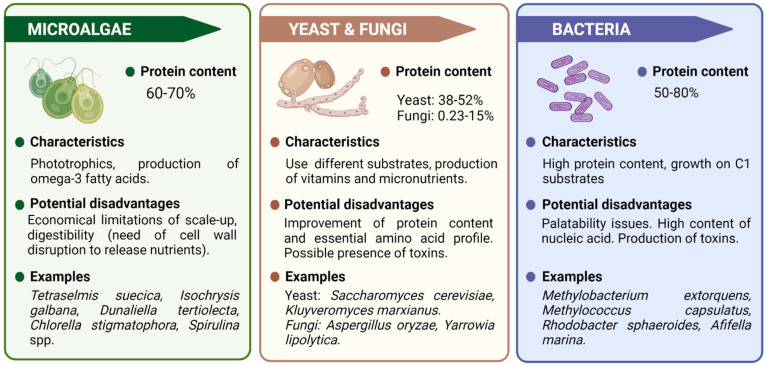
Microorganisms used in SCP production and its main characteristics.

In the same way, plant-based ingredients are not the best solution for aquatic species diets, as it might compete with human feed production; e.g., the use of land to feed fish instead of human directly. There also are issues with digestibility of plant-based ingredients by carnivorous species, or the presence of antinutritional factors, among others [21]. Among the alternatives suggested to replace fish protein in aquaculture diets, the utilization of single-cell protein (SCP) stands out. SCP refers to protein extracted from pure or mixed cultures of microorganisms such as microalgae, yeast, fungi, or bacteria (Figure 1), and it can be used as a substitute for the conventional protein sources destined for human and animal consumption [22]. Regardless of the microorganism used, SCP has important advantages over traditional sources of proteins as it requires a shorter production time, less use of land, and its production should not be affected by weather conditions [23,24]. Therefore, this review is aimed to show the potential that the use of SCP may bring as alternative protein source as well as the several solutions it may provide over a myriad of products and production tactics.

## 2. Single-Cell Proteins Production Systems

### 2.1. Microalgae

Microalgae are considered a potential source of feed due to their chemical composition, which includes proteins, essential fatty acids (eicosapentaenoic and docosahexaenoic acids) [25], and several bioactive compounds (pigments and phenols) [7]. Focusing on their protein content, it significantly varies, depending on the microalgal species, from 0 to 60% weight, with a mean value of 34% [4]. Hence, choosing the species with a proper protein composition from a nutritional point of view is vital for the process to be profitable and to be able to fill the so-called “protein gap”, which is the deficit between demands and supplies of proteins worldwide [26]. The most commonly used species are marine species, due to their chemical composition and abundance. Some of the most relevant are *Isochrysis galbana, Tetraselmis suecica, Dunaliella tertiolecta*, and *Chlorella stigmatophora* [27,28,29], which have amino acid profiles remarkably similar and comparable to the reference proteins (i.e., comparable to egg or human milk protein), but with a low content of methionine and cystine and a high content of lysine. In all of them, the total nucleic acid content is less than 7% of the dry weight, a value lower than in yeasts or bacteria [28]. At present, several studies have used microalgae to optimize different diets (Table 1). For example, *I. galbana* at different concentrations (25% or 100%) was evaluated as feed for goldfish (*Carassius auratus*) larvae. The highest survival rates were achieved with the control diet and the diet containing microalgae biomass as a substitute of 25% of fish protein hydrolysate [29]. *Tisochrysis lutea* and *T. suecica* freeze-dried biomass were evaluated as European sea bass (*Dicentrarchus labrax*) feed. This diet had no adverse effects in growth performance and feed conversion efficiency. However, a slight decline in dry matter, protein, and energy digestibility was observed in response to graded levels of dietary microalgae biomass, which can be compensated for by increased feed intake [30]. Another study that evaluated the use of *Spirulina pacifica* as protein supplement showed its capacity of increasing body weight when compared against the basal diet [31]. The best results in terms of weight gain, protein efficiency index, and food intake were obtained with diets having 5% *S. pacifica*. Moreover, concentrations of 7.3% of *S. pacifica* combined with a relatively high content of soybean flour replaced up to 15% of the protein in parrotfish (*Scaridae* spp.) diet [32]. In addition, this alga was shown to provide color to fish meat. For example, rainbow trout (*Oncorhynchus mykiss*) supplemented with different concentrations of *S. platensis* (0, 2.5, 5, 7.5, and 10%) displayed fillets with a higher coloration due to carotenoid deposition in the tissue [33]. Another widely used alga for replacing fish protein is *Haematococcus pluvialis*. The genus *Desmodesmus* can be used up to concentrations of 20% with comparable results and without any adverse effect on Atlantic salmon (*Salmo salar*) growth [34]. *Scenedesmus almeriensis* could replace up to 40% of fishmeal in fish diet, though growth was on the lower limit of acceptable yield [35]. Other successful examples of alternative diets include the use of the so-called “green water meal”. This diet is based on the use microalgae that have grown in the green water employed for tilapia (*Oreochromis* spp.) production. The composition of this microalgae mixture, which is unknown, was shown to be a suitable fish meal replacement in the diets of juvenile Pacific white shrimp (*Litopenaeus vannamei*) [36]. Shrimps (*Litopenaeus vannamei*) fed with different supplements (3, 6, 9, and 12%) of this alga increased their reddish color to its high content of astaxanthin. Therefore, these algae can be a valuable alternative protein and pigmenting ingredient in shrimp feed [37]. *Chlorella* spp. is also commonly studied. Its nutritional effect was studied in Prussian carp (*Carassius gibelio*), showing that optimal results can be obtained with algal supplements of 0.8 and 1.2%. Between these concentrations, an increase in growth performance, immune response, and digestive enzyme activity was observed [38]. Similarly, red cherry shrimp (*Neocaridina davidi*) fed with different concentrations (8–10%) of *Arthrospira platensis* showed better growth and reproductive rates [39].

Based on all these results, microalgae can be considered a potential alternative source of protein in aquaculture. However, it is necessary to follow some guidelines, since a diet should not be based exclusively on microalgae due to its digestibility. Not all microalgae species are suitable for certain aquaculture species; thus, it is necessary to analyze each specific case. For example, a comparative study about digestibility of different species (*Nannochloropsis* sp. and *Desmodesmus* sp.) showed that *Nannochloropsis* spp. was more digestible for salmon and that its digestibility was increased by extrusion processes according to higher values of digestibility of ash, dry matter, and protein than that of non-extruded diet [65]. This increase in digestibility might be due to the process of denaturalization in the extruder, which is believed to improve their digestibility by exposing the molecules to more enzyme access sites [66]. Therefore, more research should be carried out to evaluate the growth potential of microalgae and determine the factors that affect their effectiveness [67]. In general, it is necessary to optimize the production of microalgae to increase their protein yield and develop specific studies to assess the feasibility of their inclusion in fish diets since they may provide a partial replacement of animal proteins and so be doubly beneficial—economically and environmentally.

### 2.2. Yeast and Fungi

This group can be divided into unicellular (yeast) and filamentous fungi. Although both groups have been applied in aquaculture, most studies have focused on the use of yeast. Yeasts species routinely used in aquaculture are considered the major protein-rich ingredient in aquafeeds, with crude protein contents of 38–52% dry matter [68]. These species include *Saccharomyces* *cerevisiae, Cyberlindnera jadinii, Kluyveromyces marxianus, Blastobotrys adeninivorans, Wickerhamomyces anomalus*, *Aspergillus* spp., and *Fusarium venenatum* [68]. Some novel strains for protein replacement include synonymous subspecies and asexual forms of *K. marxianus*, such as *K. fragilis, K. lactis, K. bulgaricus, Candida kefyr*, *Candida pseudotropicalis*, and *Candida utilis* [23,55]. Regarding filamentous fungi, the content of nitrogen significantly varies between species [59,69], ranging between 0.23 and 15% dry matter [70]. Some filamentous fungi species commonly used in aquaculture include *Hansenula jadinii, Yarrowia lipolytica* [23,55], *Aspergillus oryzae, Neurospora intermedia*, and *Rhzopus oryzae* [71,72].

Several studies have evaluated the supplementation of yeast in aquaculture feed (Table 1). For instance, *C. utilis* and *K. marxianus* were reported to be suitable protein sources in diets for Atlantic salmon (*S. salar*). These two species could replace up to 40% of high-quality protein without adversely affecting growth performance, digestibility, or nutrient retention. However, *S. cerevisiae* reduced the growth performance and nutrient amounts [55]. This is in accordance with other results that allow to conclude that *S. cerevisiae* alone is not suitable as SCP. In another study, 40 and 60% replacement of fish meal protein with a mixture of *W. anomalus* and *S. cerevisiae* modified the gut microbiota of rainbow trout, while 20% replacement and diets with only *S. cerevisiae* had little or no effect [73]. Lower concentrations (1–4% *S. cerevisiae* and dietary yeast hydrolysate) can also improve growth performance, modulate intestinal microbiota, enhance innate immunity, and strengthen resistance of ammonia nitrogen stress [72,74]. Another study in which shrimp were fed with this yeast and soy showed a significantly higher weight gain, as well as a higher availability of nutrients than the fishmeal diets [54]. Recently, a study showed that *C. utilis* could be an alternative source of protein in the diets of *S. salar,* since it increases the growth taxa of the fish without obvious adverse effects on gut health [57]. The administration of this yeast supplement to *Litopenaus vannamei*, in increasing dietary proportions (0, 7.5, 15, 30, 60, and 100%), had statistically similar growth and survival rates; hence, it demonstrated that *C. utilis* can be used as an SCP dietary ingredient [75]. Other yeast like *Rhodotorula mucilaginosa* can also be used as an SCP, as seen in a study conducted with juvenile Nile tilapia. According to the results, a 1% supplementation of this yeast improved the growth rate while reducing the feed-conversion rate [76]. The use of these species has beneficial effects on fish growth, but also other positive health effects, including immunostimulant activity, antibacterial activity for disease prevention, and improvement of antioxidant defenses [57,59,76].

Regarding the SCP of filamentous fungal origin, they are well digested by fish [77], which facilitates their incorporation into aquaculture feeds. However, the number of studies available with this raw material is scarce (Table 1). *Aspergillus oryzae* was added in the diet of Nile tilapia (*O. niloticus*) at different regimens (every day or alternate days). Results confirmed that any feeding regimen provided similar outcomes with respect to growth, digestive enzyme activity, and intestinal histomorphology [78]. *Yarrowia lipolytica* has been utilized as an alternate source of n-3 fatty acids in salmon aquaculture [79] The apparent digestibility of these lipids can be increased by cell rupture processes from 26% (in unwashed biomass) to 32% (in washed biomass) to 76% for eicosapentaenoic acid, and so it prompted an increase in the n-3 lipid content in *S. salar* [80]. Moreover, supplementation of *Y. lipolytica* biomass (3–7%) for 35 days to Nile tilapia had growth and immunostimulatory effects according to the levels of lysozyme, myeloperoxidase, and nitrite/nitrate content in the blood of animals [81]. White shrimp fed with *Y. lipolytic* also showed an increase in the immune parameters in comparation to glucan and the control diets [82]. Therefore, even though yeasts have been studied as SCP for protein replacement in fish feeding, filamentous fungi represent another alternative to continue exploring.

### 2.3. Bacteria

Bacteria are also important SCP producers, characterized by their high protein content (up to 80% weight) and proportion of essential amino acids along with vitamins, phospholipids, and other functional molecules. Moreover, these microorganisms can be produced using a wide variety of feeds, which increases their production and applicability [23]. Furthermore, the ease production of SCP through aerobic fermentation processes broadens its utilization in aquaculture [83]. The most used species to produce SCP are *Methylobacterium extorquens, Methylococcus capsulatus*, *Rhodobacter sphaeroides*, *Afifella marina,* and *Corynebacterium ammoniagenes,* among others. Bacteria have been tested mostly on shrimp species, although some works have studied their suitability in fish. The effectiveness of bacteria for feed in aquaculture has been repeatedly proven in various published works (Table 1). In one of them, the bacteria *M. extorquens* was supplied to white shrimp (*L. vannamei*), smallmouth grunt (*Haemulon chrysargyreum*), and Atlantic salmon (*S. salar*) at variable concentrations. The best results in terms of acceptability, growth, and survival rate were obtained with 30%, 100%, and 55% supplementation for grunt, shrimp, and salmon, respectively, and pointed to this bacterium as a potential substitute for SCP in aquaculture feeds [83]. The suitability of *M. extorquens* was also evaluated in rainbow trout (*Oncorhynchus mykiss*) to replace a percentage of the soybean meal (5–10%). Data displayed that a 10% SCP diet improved fish survival even when weight gain was slightly lower. The authors claimed that the addition of palatability-enhancer ingredients may improve the obtained results [84]. Another bacterium, *M. capsulatus,* has been supplied to *S. salar, Oncorhynchus mykiss*, and *Hippoglossus hippoglossus*, with promising results [85]. In the case of salmon, the effect of different proportions (0, 4.5, 9, 18, or 36%) of bacteria in the diet was analyzed. Lower concentrations increased the branchial and/or renal nitrogen and energy losses and the energy spent on activity and maintenance [63]. In addition, these types of bacterial diets avoid intestinal problems caused by other types of foods (e.g., enteritis induced by soy flour). Therefore, *M. capsulatus* is a suitable protein source for salmonids [86]. Other species, such as *R. sphaeroides* and *A. marina*, also have been used to supplement white shrimp, this time in a 1:1 ratio at different concentrations (1, 3, and 5%), for the elaboration of feed. Diets with the lowest concentration of bacteria (1%) showed a higher growth performance and survival rate (85%), proving to be a potentially effective source of SCP in shrimp [87]. Another bacterium used with white shrimp is *C. ammoniagenes,* commercialized as PROTIDE (CJ BIO, South Korea). The best performance of the product, in terms of final weight, weight gain, specific growth rate, and feed conversion ratio, supplied at different concentrations (0, 10, 20, 30, and 40%) to shrimp, was observed in the 0 and 20% diets [88]. For all concentrations under study, there was an increase in crude protein in white shrimp [88]. Similar results (increase in growth performance) were obtained with black tiger shrimp (*Penaeus monodon*) supplemented with a microbial bioactive (Novacq™; CSIRO, Australia) [89]. Actually, some studies have focused on the use of purple phototrophic bacteria (~60% crude protein DW), as feed for salmonids, carnivorous marine fish, and shrimp. However, it is not yet possible to find applications for this product at an industrial level since it is in the early stages of development [90]. Therefore, even though to the date several species of bacteria have been proven to be safe and efficient to replace the animal protein content in aquaculture species’ diets, it is worthy to keep evaluating more species to disclose their potential as feed.

Besides, SCP competitiveness of the organism under study can be increased in terms of production cost, nutrition, and functionality by genetic and microbial engineering. Moreover, there is an increasing demand to produce SCP products that include multifunctional ingredients rather than just protein. This is the case of KnipBio Meal (KnipBio, USA) [91].

## 3. Opportunities to Meet Circular Economy: Revalorization of Industrial Sub-Products as Substrate for SCP Production Systems

**Figure 2:**
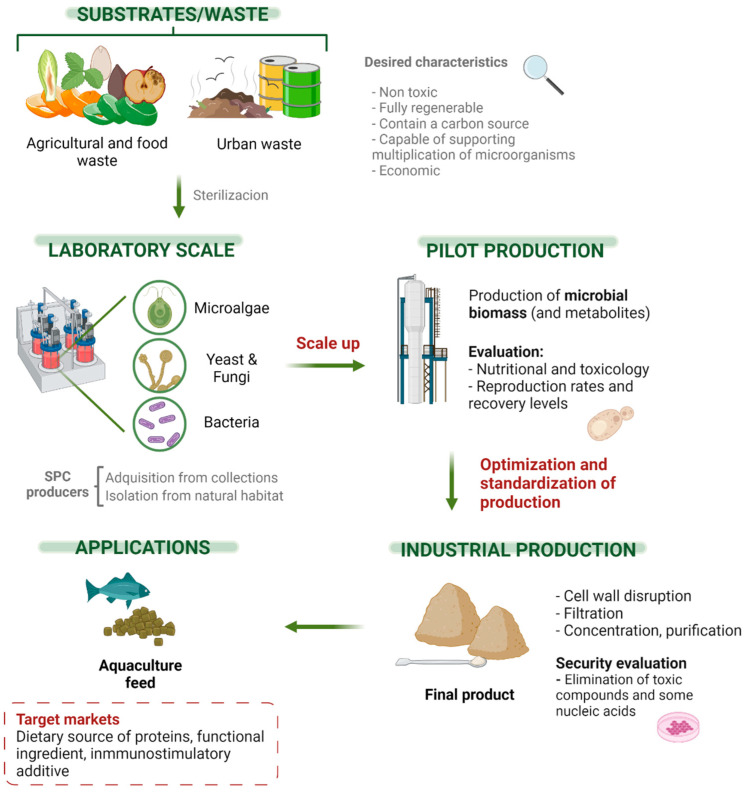
Optimal single-cell protein (SCP) production processes, integrating circular economy approaches.

Increasing public awareness of environmental and ecological factors has led consumers to increasingly demand more sustainable products. Different strategies, ranging from local production to more innovative concepts such as the circular economy, already have been developed to achieve sustainable items [92]. A circular economy is a production and consumption model that implies sharing, renting, reusing, repairing, renewing, and recycling existing materials and products as many times as possible to create added value. In the food sector, this would be fundamentally reflected in reducing both the entry of raw materials and production of waste, closing the economic and ecological flows of resources [93]. Therefore, the application of this concept to aquaculture is of great interest since it will make possible to satisfy, in a more sustainable way, the need for animal protein production. Recycling diverse types of wastes, such as those from agriculture, urban, and food, can provide a carbon source for SCP production [94,95] (Figure 2). Therefore, this practice would improve waste management [96]. In fact, in the regulation EC 2008/98, European Member States are encouraged to apply the waste hierarchy. For example, in Article 22, the separated collection of bio-waste aimed for composting and digestion, treating bio-waste in a way that fulfils an important level of environmental protection, is contemplated [97]. This sustainable use of natural resources has been promoted in the European Union since 2005 [98]. In this sense, an aquaculture feeding model based on the utilization of SCP satisfy all these demands, since it reduces the water footprint, the emission of greenhouse gases, land use, and minimize the destruction of biodiversity (Figure 2).

Depending on the type of waste, a specific range of value-added products may be recovered, conducting a revaluation of residues. However, this is an underdeveloped technique that requires additional research to improve its performance and widen its application fields (production of enzymes, essential oils, bioactive compounds, building block chemicals, and SCP) [99]. Therefore, the use of waste for SCP production could be an eco-friendly solution for protein demand and for waste management, as it can convert residues into food or feed [100]. In addition, the use of these biodegradable waste would reduce the production costs of SCP [99]. However, not all type of waste will be suitable for use as a substrate to produce SCP. The substrate/waste must be non-toxic, abundant, fully regenerable, non-exotic, cheap, and capable of supporting rapid growth and multiplication of the organisms, which would result in a high-quality biomass [100]. Moreover, all waste used must be a carbon source [101]. Currently, most of the available works analyze the type of organism used to produce SCP. The reason is that accessing different strains of microorganisms is simple; however, the availability of waste products is specific to each local economy, and thus highly variable options may be adopted. Generally, substrate/waste can be classified into four large groups: (1) sources rich in mono- and disaccharides; (2) sources rich in starch; (3) sources rich in structural polysaccharides; and (4) sources rich in proteins or lipids [101]. The choice of one sub-product over another will depend on local availability, waste pretreatment costs, transportation costs, and performance of the substrate in terms of biomass and protein production. In addition, the long-term process efficiency, the methods for separation of the biomass from the medium, the methods available to extract SCP, and methods to eliminate impurities are also factors taken into account in the choice of sub-product [101]. Hence, each group of industrial waste has its own advantages and disadvantages when used as a substrate to produce SCP [102]. For example, the utilization of polymer-rich sub-products is problematic, primarily due to the extensive pretreatments these wastes require before efficient SCP fermentation can take place [99]. Sub-products can also be classified based on their origin: food (including agricultural residues) or urban (Figure 1 and Table 2).

### 3.1. Food and Agricultural Sub-Products

Globally, approximately a third of all food produced for human consumption is lost or wasted [111,112]. According to the UNEP (United Nations Environment Program) Food Waste Index 2021, around 931 million tons of food waste was generated in 2019. Of this quantity, 61% was from households, 26% from the food service industry, and 13% from retail [113]. In the European Union, around 88 million tons of food waste are generated annually, which is equivalent to a lost/waste of 20% of the total food produced. This quantity has associated costs estimated at 143 billion euros [3]. This quantity will continue to increase as different models establish that solid waste will increase approximately 7.5% per year [114]. Agriculture also generates considerable amounts of vegetable residues. In many cases, these agriculture residues are untapped, and therefore their revalorization as a substrate to produce SCP is of interest. Furthermore, they are well characterized in terms of chemical composition and show high contents of lignocellulosic biomass (cellulose, hemicellulose residues) that may be appropriate as SCP substrates [115]. Lignocellulose (30–56% cellulose, 3–30% lignin, 10–24% hemicellulose, and 3–7.2% protein) has limited applicability in other fields due to its low digestibility and low protein content, which makes it unsuitable for animal feed [116]. Some agricultural wastes rich in lignocellulose are wood chips, sawdust, or ears of corn. However, the most routinely used for SCP production are starch, molasses, fruit and vegetable waste, as well as unconventional substrates such as agro-industrial wastewater [117,118]. Studies revalorizing food and agricultural sub-products for SCP production have been collected in Table 2. For example, orange pulp or brewer’s spent grain can be used to culture *S. cerevisiae,* achieving a protein yield of 38.5%. Similarly, these residues, together with whey and potato pulp, allowed to obtain a protein yield of 33.7% from *K. marxianus* [103]. Three agro-waste streams ((1) orange pulp, juice, and peel; (2) lemon pulp, juice, and peel; and (3) corn stover effluent) were used as substrate for *Rhodococcus opacus* production, which is used as SCP for aquafarming and livestock [107]. In order to increase the production of SCP from lignocellulose, it is commonly required to perform a previous step of hydrolyzation, which may be regarded as a drawback because it is time consuming [119]. This inconvenience can be solved by using cellulolytic microorganisms [120]. For example, cucumber and orange peels have been used to produce biomass by submerged fermentation using *S. cerevisiae*. These substrates were suitable due to the high degree of hydrolysis achieved, which resulted in higher yields of proteins with cucumber peels (53.4% instead of 30.5% of orange peel). Moreover, it was possible to increase the protein production by adding glucose to the hydrolyzed medium [105]. Other substrates used for the production of SCP of *S. cerevisiae* include the combination of dried potato skins with carrot skins [104] or discarded whole foods composed of mixtures of fruits and vegetables [106]. Other microorganisms that can be produced with this type of substrate include purple phototrophic bacteria or strains of *R. opacus* bacteria [107].

Hence, food and agricultural residues can be reutilized as a potential source of ingredients to develop culture media for diverse types of SCP microorganisms. This approach would recycle biomass volumes, avoiding its management costs, reducing its environmental impact, and giving an added-value product.

### 3.2. Urban or Industrial Wastes

In global terms, in 2020, global waste generation was estimated at 2.24 billion tons of solid waste, amounting to a footprint of 0.79 kg per person per day according to the latest report by the World Bank [121,122]. These values are expected to continually increase due to rapid population growth and urbanization, which lead to estimations of an increase in annual waste generation of 73% from the 2020 levels to 3.88 billion tons in 2050 [121]. The amount of urban and industrial waste differs between countries due to differences in economic level. In the USA, it is estimated that the total generation of municipal solid waste (MSW) in 2018 was 292.4 million tons, equivalent to 2.2 kg per person per day. Of the MSW generated, approximately 69 million tons were recycled and 25 million tons were composted, equivalent to a 32.1% recycling and composting rate [123]. These types of residues includes petroleum by-products, natural gas, ethanol, methanol, and human and animal excreta [117,118]. The main petroleum fractions used in SCP are hydrocarbons, especially those containing C12–C22 [124]. Urban wastes are a good choice in terms of biomass and gas productivity. Moreover, biogas derived from organic wastes generates methane, which can be used as a carbon source for SCP production on a larger scale to lower the total cost of production and reduce dependence on fossil resources. Nitrogen deficiencies in this system can be overcome by direct addition of pasteurized centrifugal filtered digestate or by adding ammonium electrochemically extracted from the digestate [108]. Furthermore, the microbiological treatment of biodegradable waste materials ensures the neutralization of harmful substances and allows a reduction in environmental pollution [125]. Some examples of SCP production using urban waste have been compiled (Table 2). A study evaluated the possibility of valorizing urban biowaste by combining anaerobic digestion and SCP production, feeding a mixed culture of methanotrophs with raw and upgraded biogas. It was seen that the yield of SCP in methane varied from 0.59 to 0.76 g of cellular dry weight/g CH_4_, also demonstrating that biogas is a good substitute for natural gas. In addition, the SCP produced was rich in essential amino acids, making the biomass produced comparable with other sources of proteins [108]. Biogas derived from the anaerobic digestion of sewage sludge and the discarded effluent can be used as a nutrient source to produce SCP using methanotrophic bacteria (mainly *Methylomonas* spp. (56.26%) and *Methylophilus* spp. (24.60%)). The resultant dried biomass had a protein content higher than 41% w/w of dry weight, with significant concentrations of essential amino acids such as histidine, valine, phenylalanine, isoleucine, leucine, threonine, and lysine [109]. *Methylococcales* and *Methylophilales* were also produced with municipal solid waste showing a variability in the protein content between 8 and 20% w/w of dry weight [108]. *Rhodopseudomonas* sp. can be used and produced in wastewater treatment, obtaining a crude protein content of 60.1% w/w of dry weight, containing all the essential amino acids [110].

Hence, wastes considered to have a strong impact on natural ecosystems, such as urban and industrial petroleum-based wastes, can be reduced by their reutilization in the process of SCP production, which provides an alternative recycling strategy. Thus, SCP can reduce the health risks through improving waste management and reducing exposure to pollution and a wide range of harmful substances (i.e., by regulating the use and disposal of chemicals, or by substituting hazardous chemicals with more benign substances).

## 4. Process Application

### 4.1. Design of a SCP Production Plant

In the design of an SCP production plant, it is necessary to carry out optimization of the cell growth and co-product yields, to assess the economic viability of the project (Figure 2). One of the most determining parameters is the access to raw material or substrate, which represent a factor specific to each region and very variable in terms of costs among different evaluated areas (availability, transport, preservation, etc.). Therefore, by-products of local production are usually chosen [126], being in most cases raw materials rich in lignocellulose [127]. The type of substrate will determine the microorganism to produce. The choice of microorganisms will also depend on the process and the desired quality of biomass. It is important to select those microorganisms that (1) are not pathogenic for plants, animals, or humans; (2) have good nutritional value; (3) do not have toxic compounds; and (4) are legally accepted as food or feed [128]. In most cases, combinations of specific residues and organisms maximize yield production (e.g., waste from the wine industry or stillage containing *Chaetomiun cellulolyticum*; citrus peel residues with *Fusarium culmorum, Geotrichum candidum*, and *Trichoderma viride*; banana and cane bagasse using *Saccharomyces cerevisiae*) [129]. Moreover, to maximize the efficiency of this process, both from an economical and an environmental point of view, the CO_2_ produced during the fermentation may be recycled as a carbon source that can be fixed by photosynthetic organisms such as *Scenedesmus* spp. This procedure would yield algae biomass and O_2_ [130]. Biomass can further be used as a feeding source and O_2_ can be further recycled for aerobic fermentations. Another crucial step for SCP production is the sterilization process, which can eliminate inhibitors of bacteria growth, thus avoiding contamination of the mixture [115]. The scale of the fermenter is also of significant importance to the economic viability of SCP production as there is an empirical relationship between cost and scale of production [77]. Optimal sizes allow high biomass production, high O_2_ transfer rates (anaerobic organisms can also be used in bioreactors), and high respiration rates, which, in turn, increase metabolic heat production. Controlling this increase in temperature is essential for microorganisms to remain practical and reproducible. Thus, it is necessary to use efficient cooling systems, which entails a significant energy expenditure [115]. Continuous system operations have been shown to be the most profitable [77]. Therefore, the most important economic factors are the investment in equipment, energy expenditure, operating costs, waste, safety, and availability in the market [115]. Based on these principles, a study estimated that in the case of fungal SCP, the total cost of the raw material accounts for 62% while the production process involves 19% of the costs. In this sense, industrial sectors keep evaluating different substrates or raw materials that may reduce their economic impact [77], but more studies are necessary. Another economic disadvantage of this production system is the drying and concentration process that may become energetically excessive and economically expensive, so green and more efficient energy sources are always under assessment.

### 4.2. Food-Safety of SCP

SCP must not only have a nutritional value, but also be safe according to toxicity tests to be marketed as a food product for animals or humans. Tests carried out must include assays of acute short-term toxicity with several different species of laboratory animals, followed by extensive and detailed long-term studies [115]. The biggest toxicological problems lie in the presence of toxins (e.g., mycotoxins in fungi or cyanotoxins in cyanobacteria) or unwanted compounds (e.g., heavy metals) accumulating during growth, as well as the content of nucleic acid (especially important for human consumption) [131], as these may cause, for example, allergy symptoms or kidney stones [132]. Therefore, the selection of the species needs to be carefully performed, as well as control the conditions in which the production and formulation of the product is carried out [131]. Nowadays, it is possible to find patents on the market that have passed all these tests. Some examples intended for human consumption include Quorn™ mycoprotein (Marlow Foods Ltd., Billingham, UK) [133], or the species *Y. lipolytica*, which can be marketed as Toprina (Nucelis Inc., San Diego, CA, USA) (Table 3). However, it should be noted that this species can cause rare opportunistic infections in severely immunosuppressed or ill people with other underlying diseases or conditions. These infections can be treated effectively using common antifungal drugs and, in some cases, even disappear spontaneously, which is why *Y. lipolytica* is considered a safe organism to use [134]. However, although the safety and nutritional value of SCP have been demonstrated in commercialized species, these products are sometimes rejected by consumers due to the associated poor opinion of consuming microbes and related subtle psychological, sociological, and religious implications [119]. Besides, consumers’ rejection can be also prompted by the organoleptic properties related with the presence of microorganisms [135].

### 4.3. Benefits and Drawbacks of Using SCP as Aquaculture Feeds

The major advantage of SCP production is the high throughput in terms of protein production [148], which mainly vary between 50 to 70% protein in dry weight, although it can be higher than 85% in some species of *Clostridium* [138]. The appreciable nutritional value of SCP is also due to its high vitamin content [149]. Moreover, under optimal conditions, these microorganisms have a fast population-doubling rate (5–15 min bacteria or yeast; 2–6 h microalgae and fungi), with a better feeding efficiency than mammals (1 kg yeast cells—2 kg glucose; 1 kg beef—18 kg cereals) [150]. Furthermore, many SCP formulations have shown positive effects on immunological, microbiome, and inflammatory responses in different species (Atlantic salmon, gilthead seabream, and rainbow trout, among others) [151]. All these benefits can be increased with genetic manipulation as microorganisms have an easier genetic improvement and transfer than higher animals and plants [152]. In addition, different studies support that the production of SCP in food and feed would exert positive effects on the environment (see Section 1 for further details). Additionally, SCP can be produced at any time of the year since they are not dependent on seasonal and climatic variation [48].

However, SCP also has some drawbacks. One of its main limitations is the presence of antinutritional factors, including the high content of nucleic acids, the presence of cell walls, or allergic compounds. Important levels of nucleic acids have been reported to increase the uric acid concentration in the serum, with the consequent formation of kidney stones. In turn, cell walls cannot be digested by herbivore animals [150]. Additionally, some microorganisms used in SCP can generate toxic substances, such as mycotoxins or cyanotoxins. Even though. many of these antinutritional factors can be eliminated by using different physical and chemical methods during processing; however, this additional processing rises the cost of production [153]. On the other hand, microorganisms that form SCP must be inactivated before being supplied as feed in aquaculture, otherwise they may cause diseases and generate unwanted colors and/or flavors, as well as palatability problems [150]. Another drawback of SCP is the aminoacidic proportions, especially lysine and methionine, which are unbalanced compared to fishmeal, so that the palatability of SCP is not as good as fishmeal for aquatic animals. For example, in fungi, the SCP lysine content is typically high, but the methionine content is relatively low [132]. Finally, it is important to highlight that not all SCP formulations have the same potential in aquaculture.

In general terms, the main industrial limitation of SCP is economic. To reduce their high cost of production, most of the current strategies carried out are focused on the use of intensive fermentation systems and a reduction in input cost, while maintaining the quality and increasing productivity [151]. An example of these approaches is the development of biofloc formulations. Bioflocs are heterogeneous aggregates of suspended particles and a variety of microorganisms associated with extracellular polymeric substances [154]. The basis of biofloc technology is the mass production of in situ microorganisms, which is credited for maintaining good water quality, increase culture feasibility by reducing the feed conversion ratio and feed costs, provide biosecurity, and are capable of sequestrating greenhouse gasses [155]. In this way, several microorganisms with potential applications can be produced simultaneously and with a positive effect on the environment.

### 4.4. Legislation of SCP

The legal frame of SCP is complex due to the variability of sources from which the biomass can be obtained. Moreover, SCP also has diverse applications, being each of them subjected to different regulatory frames. In most cases SCP is destined to be used as food or feed, but it is also common to find them as additives (e.g., as colorants). In all cases, the protein of the microorganisms is still present in the final product, which limits the extent to which they are added and their value as SCP.

Furthermore, the legislation differs notably among countries or political/economic areas, being only consistent and unified in Europe. In 2013, a detailed review compiled different specific regulations related to feed and feed additives from diverse economic and political areas such as Brazil, Canada, China, Europe, Japan, South Africa, and United States [156]. Among the remarkable results presented in this work, it should be noted that not all animals are considered equal in all regions, which is why pet food is regulated as food in some areas, but not others. Therefore, authorization is needed before the sale of new feed or additives. In all cases, it will be necessary to conduct a toxicological study prior to marketing a product, to prove its safety.

### 4.5. Environmental Impact

Circular management of carbon and nutrients is at the basis of future environmental sustainability and global food safety. Therefore, production of SCP might have a positive environmental impact as it can be a better strategy than other currently available technologies (e.g., anaerobic digestion) for reducing food waste [130], or manmade waste streams management, which are harmful if they are released into local environments [125]. However, to achieve a sustainable process, it is necessary to conduct a careful evaluation of each case and develop a process that couple anaerobic digestion and thermochemical gasification. This combination of techniques allows to convert biowastes into clean gaseous substrates (e.g., H_2_, CH_4_, CO_2_, CO, NH_3_, and P_2_), which are used for the fermentative (aerobic or anaerobic/aerobic) production of safe SCP [157]. Moreover, by using these wastes for the production of SCP, the neutralization of harmful substances is ensured, and environmental pollution is reduced, resulting in a value-added product [125]. In fact, the combined production of SCP and biochar can capture and store up to 2.33 Gt CO_2_-equivalents per year, which represents approximately 50% of the Paris Agreement target on annual carbon sequestration [157]. Understanding the environmental benefit of SCP production has led to a renewed interest in this type of product, also helped by the development of a more suitable production process [158].

In addition, the use of this type of protein supplement would replace or reduce the traditional and unsustainable sources of protein supplements (e.g., soy flour). These protein sources are considered inefficient and unsustainable due to their low conversion rates: for every 162 units of nitrogen apported to the production system (fertilizers, manure, biological fixation), 96 are lost due to volatilization and runoff processes [159]. This rate would be drastically increased with the production of SCP [160] as this system could recycle up to 18.5 million tons of nitrogen per year (∼8% of current nitrogen losses) and 6.5 million tons of phosphorus per year (∼25% of annual phosphorus fertilizer production) [157]. Nevertheless, earlier and detailed evaluations are required to minimize the environmental impact of SCP against other alternative protein production systems. Few studies have reported that cultured and plant-based meats have lower eutrophication potential than SCP [161], whereas other works point to the environmental impact that the indirect land-use changes for soybean production may have [162]. Currently, it is estimated that the global annual production of SCP is 606 million tons, which would be thrice the soybean meal protein production [157]. Therefore, complementary studies on the economic and environmental viability of each of the production systems are necessary. To be economically practical, it is necessary to calculate the minimum SCP biomass value necessary for food waste management to be a net positive enterprise. From an environmental point of view, it should be compared with food waste management practices [160]. In a recent study, the production of different protein supplements was compared; it was concluded that SCP production has less environmental impact than soybean meal, but its use from an economic point of view is more limited [160]. This greater impact from an economic point of view is due to the greater demand for thermal and electrical energy [162]. On the contrary, comparing SCP with the production of fishmeal, a greater environmental limitation is observed, but an economically more favorable process [160]. To help minimize the potential negative effects of SCP production on the environment, the use of autotrophic microorganisms has been reported to maximize energy recovery. This maximization is what future research should focus on, taking into consideration a heat demand approach [162].

## 5. Conclusions

The imminent increase in population worldwide leads to a parallel increment in the demand for protein destined for human consumption. Aquaculture may be a productive system to meet this increasing demand; however, it requires the application of innovative management designs to become more sustainable by using renewable resources. The application of the principle of a circular economy to aquaculture may solve its main drawback: the requirement of excessive animal proteins in the diets of the animals. In this way, underused waste from industrial sectors such as agriculture, food, or urban areas can serve for the development of an innovative source of protein used for feeding aquaculture animals. The main purpose is to change aquaculture diets through the substitution of fish and vegetable meals for SCP preparations obtained from bioreactors fed with various sub-products. These SCP-based meals have the potential to provide aquaculture with sustainable and renewable food ingredients to compensate for deficiencies in plant meals and reduce the need for fishmeal in diets. Nowadays, few SCP flours are commercially produced. In addition, numerous studies have showed the positive benefits in different aquaculture species of fish (such as salmon, trout) and shrimp fed with SCP-based diets, such as improvements in survival and growth performance, modulation of intestinal microbiota, enhancement of innate immunity, and strengthened resistance against stress. Regardless, there are still challenges for scaling up SCP production, processing, and the economics of a commodity. However, in the few last years, particularly important advances have been made with respect to the search for new strains, diverse types of substrates, the development of new processes, and successful tests in fish species, which is highly encouraging for SCP products. Therefore, SCP might be a potential supplement of proteins for both animals and plants in an economic way as they can be produced at any time of the year and from various cost-free substrates, almost without a negative impact.

## Figures and Tables

**Table 1:** Examples of several SCP-based diets used as feed for different species in aquaculture: protein intake and digestibility assessments.

Feed	Protein Content (%)	Species	Dose	Digestibility (%)	Process	Ref.
**Microalgae**						
*Nannochloropsis* spp.	39.3	*S. salar*	30% diet	72.0	Extrusion	Ref. [40]
*Nannochloropsis gaditana*	39.3	*Oreochromis niloticus, Clarus gariepinus*	30% diet	72.4; 74.7, respectively	Dry	Ref. [41]
*Desmodesmus* sp.	37.3	*S. salar*	30% diet	67.0	Extrusion	Refs. [34,40,42]
*Schizochytrium* sp.	9.4–42.5	*O. niloticus*	Fish oil	82.0	Extrusion	Refs. [43,44]
*Chlorella vulgaris*	17.9	*O. niloticus, C. gariepinus*	30% diet	80.7; 80.9, respectively	Dry	Ref. [41]
*Scenedesmus* sp.	48	*Onchorhynchus mykiss*	5% diet	No data	Pellets	Ref. [45]
*Scenedesmus dimorphus*	48	*O. niloticus, C. gariepinus*	30% diet	67.0; 68.3, respectively	Dry	Ref. [41]
**Fungi**						
*Aspergillus niger*	17–50	*Penaeus vannamei*	50–60% diet	80.7; 81.7, respectively	Dry	Refs. [46,47,48]
*Fusarium venenatum*	50	*Melanogrammus aeglefinus*	No data	No data	QUORN	Ref. [49]
*Trichoderma harzianum*	34	*Danio rerio*	24.0 g/L	No data	Pellets	Ref. [50]
**Yeast**						
*S. cerevisiae*	44.4	*Salvelinus alpinus, Perca fluviatilis*	30% diet	86; 83, respectively	Dry	Refs. [51,52]
*S. cerevisiae*	44.4	*S. salar*	40% diet	73.0	Spray-drying	Ref. [34]
*S. cerevisiae*	44.4	*O. mykiss*	40% diet	91.0	Dry	Ref. [53]
*S. cerevisiae*	44.4	*L. vannamei*	30% diet	74.4	Dry	Ref. [54]
*C. utilis*	40	*S. salar*	40% diet	88.0	Spray-drying	Ref. [55]
*C. utilis*	40	*S. salar*	25% diet	No data	Drum drying	Ref. [56]
*C. utilis*	40	*S. salar*	40% diet	23.0	Extrusion	Ref. [57]
*Kluyveromyces marxianus*	9.5–12	*S. salar*	40% diet	86.0	Spray-drying	Refs. [55,58]
*Rhodotorula mucilaginosa*	No data	*O. niloticus*	1% diet	No data	Hydrolyzed	Ref. [59]
**Bacteria**						
*Arthrospira maxima*	60–70	*O. niloticus, C. gariepinus*	30% diet	81.4; 82.5, respectively	Extrusion	Refs. [41,60]
*Clostridium autoethanogenum*	83	*Micropterus salmoides*	50% diet	92	Dry	Ref. [61]
*C. autoethanogenum*	85	*Acanthopagrus schlegelii*	58.2% diet	No data	Extrusion	Ref. [62]
Biofloc	70%	*S. salar*	36% diet	88.0	Extrusion	Ref. [63]
Biofloc	No data	*L. vannamei*	30% diet	76.3	Extrusion	Ref. [64]

**Table 2:** Revalorization of industrial sub-products as substrates for SCP production systems.

Waste	Strain	Production System	Protein Yield	Characteristics	Ref.
**FOOD**					
Orange pulp and brewer’s spent grain	*S. cerevisiae*	Solid state fermentation	38.5%	Significant content of fat (12.9%).	Ref. [103]
Dried potato and carrot skins	*S. cerevisiae*	Flask fermentation	49.3%	Quantitative quality parameters comparable with casein.	Ref. [104]
Cucumber and orange peels	*S. cerevisiae*	Submerged fermentation	53.4%	Addition of glucose enhanced the protein content (60.31%).	Ref. [105]
Discarded foods (mixtures of fruits and vegetables)	*S. cerevisiae*	Simple aerobic fermentation	39.0%	Protein percentage in starting material less or equal to 8%.	Ref. [106]
Whey and potato pulp	*K. marxianus*	Solid state fermentation	33.7%	High yields of fat (25.5%).	Ref. [103]
Juice, pulp, and peel from oranges and lemons	*R. opacus*	Flask fermentation	42.0–56.9%	Protein production can be increased optimizing production conditions.	Ref. [107]
Corn stover effluent	*R. opacus*	Flask fermentation	47.0–52.7%	Protein production can be dramatically optimizing production conditions.	Ref. [107]
**URBAN**					
Organic fraction of municipal solid waste	Methanotroph mixed culture	Anaerobic digestion	20.6%	Methane derived from anaerobic digestion can be considered as carbon source for SCP production.	Ref. [108]
Methane	*Methylococcales* and *Methylophilales*	Anaerobic digestion	8.0–20.0%	Better yields at higher concentrations CO_2_ in gas.	Ref. [108]
End-products of sludge	Methanotrophic bacteria	Anaerobic digestion	41.0%	Potential alternative to partially replace soya in aquaculture.	Ref. [109]
Municipal wastewater	*Rhodopseudomonas* sp.	Anoxygenic condition	60.1%	All essential amino acids produced.	Ref. [110]

**Table 3:** Commercially available single-cell protein products.

Trade Name	Organism	Company	Country	Protein Content	Production	Other	Ref.
**Microalgae**							
Algaeon	*Euglena gracillis*	Algaeon Inc.	USA	No data	Fermentation process	β-glucan and whole cell products	Ref. [136]
Cyanotech’s spirulina	*Arthrospira platensis*	Cyanotech Corporation	USA	60%	Deep ocean water	One of the most commercialized products	Ref. [137]
ProTyton	*Clostridium* spp.	Biotech	USA	85%	Ethanol plant	Atlantic salmon, shrimp feed	Ref. [138]
**Yeast and fungi**							
Lynside^®^ Nutri	*Saccharomyces cerevisiae*	LeSaffre	USA	55.7%	Extrusion	Dried inactive yeast	Ref. [139]
Engevita™	*S. cerevisiae*	Lallemand Inc	Canada	No data	Extrusion	Dried inactive yeast	Ref. [140]
SylPro	*Candida utilis*	Arbiom	USA	>60%	Forestry by-products	Comparable to soy	Ref. [141]
Quorn™	*Fusarium venenatum*	Marlow Foods Ltd.	UK	70%	Airlift reactor	Over 17% of the global meat substitute market (2016)	Refs. [142,143]
Yarrowia flour	*Yarrowia lipolytica*	Nucelis Inc.	USA	45–55%	Agro-industrial wastes	151.2 g/L of single-cell protein at 10 L fermentation scale	Refs. [144,145]
**Bacteria**							
UniProtein^®^	*Methylococcus capsulatus*	UniBio A/S	Denmark	70%	Natural gas	Particle size of 150–200 μm	Ref. [146]
ProFloc™	Bacteria	Nutrinsic	USA	60%	Wastewater from a local brewery	Replaced up to 100% fish meal in feeds for *L. vannamei* shrimp	Ref. [143]
FeedKind^®^	Bacteria	Calysta Inc.	UK	70%	Methane	Satisfactory results in Atlantic salmon	Refs. [143,147]
String Pro	Bacteria	String Bio	India	No data	Methane	Animal feed	Ref. [148]

## Data Availability

All related data are presented in this paper. Additional inquiries should be addressed to the corresponding author.
